# Acknowledgement to Reviewers of *Biomolecules* in 2017

**DOI:** 10.3390/biom8010006

**Published:** 2018-01-24

**Authors:** Biomolecules Editorial Office

**Affiliations:** MDPI AG, St. Alban-Anlage 66, 4052 Basel, Switzerland

Peer review is an essential part in the publication process, ensuring that *Biomolecules* maintains high quality standards for its published papers. In 2017, a total of 77 peer reviewed papers were published in the journal. Thanks to the cooperation of our reviewers, the median time to first decision was 23 days and the median time to publication was 48 days. The editors would like to express their sincere gratitude to the following reviewers for their time and dedication in 2017:
Abdelraheem, Wael H.M.Liu, JunqiuAksimentiev, AklekseiLiu, YuchenAlexander, RebeccaLoewith, RobbieAngius, FedericaLopez-Aviles, SandraAquila, AndrewLoquet, AntoineBacusmo, Jo MarieLyte, MarkBailey, Shannon M.Maekawa, TakakiBeilschmidt, Lena K.Magnani, MauroBenistant, ChristineMahajan, Rakesh KumarBironaité, Daiva A.Maher, PamelaBoke, ElvanMakowski, LeeBooker, Squire J.Malberg, PerBorota, AnaMaraia, RichardBreaker, RonaldMaréchal-Drouard, LaurenceBreuker, KathrinMaster, EmmaCaldana, CamilaMateos-Timoneda, Miguel AngelCalvez-Kelm, Florence LeMatsuura, KazunoriCâmara, DiogoMcEwan, IainCampos, Guillen JuanMercer, CarolCaroli, ManuelaMihara, HisaakiChan, PatriciaMiura, SayakaChowdhury, RasheduzzamanMoir, Robert D.Chuffa, Luiz GustavoMoreno, SergioClaessen, DennisMotorin, YuriClaesson-Welsh, LenaMueller, EugeneClark, John I.Müller-Campanile, Véronique S.Cooney, RalphNakai, YumiCordo Russo, Rosalia I.Nandi, SaikatCrespo, José L.Nikonowicz, Edward P.Das, RajatNilsson, BradDasarathy, SrinivasanNoda, TakeshiDe Virgilio, ClaudioNypelo, TiinaDedon, PeterOleskin, Alexander V.Devy, JérômeOliva, RominaDierks, ThomasOrgel, JosephDinnebier, RobertOrywal, KarolinaDomínguez-Rebolledo, Álvaro EfrénPagonopoulou, OlgaDominissini, DanPan, YangangDong, Yu-HuiPardi, NorbertEriksson, UlfPatel, Yashomati M.Falasconi, IrenePhilipp, StephanFan, ChenguangPohl, EhmkeFarabaugh, Philip JamesPoulard, Coralie EugenieFarias, Jorge G.Puigvert, Jordi CarrerasFerreira, Leonardo G.Raina, SatishFicner, RalfReuter, KlausFortunato, OrazioRhee, Hyun-WooFrankel, DanielRibas, LluísGanesan, MuraliRibeiro, César Augusto JoãoGao, ShixiangRilcheva, Violeta S.Gaspari, Anthony A.Rinnerthaler, MarkGaston, KirkRizzarelli, EnricoGe, XiaodongRoelofs, JeroenGerrard, Juliet AnnRomero, DiegoGerwig, Gerrit J.Rossmanith, WalterGhosh, SantoshRowinska, MagdalenaGobeaux, FrédéricRusmini, PaolaGolinelli-Pimpaneau, BeatriceSancho-Bru, PauGoodacre, SaraSasso-Cerri, EstelaGöpel, SvenSaupe, Sven J.Gregory, Richard L.Saurav, KumarHasani, MerimaSchlimpert, SusanHe, ChuanSchoner, WilhelmHelm, MarkSchwob, EtienneHolmgren, ArneSerpell, LouiseHooda, JagmohanShigi, NaokiHori, HiroyukiSi, KausikHuang, PengSigusch, Bernd W.Huising, Mark O.Sochacka, ElzbietaImamura, SousukeSomani, AbhishekIshihama, AkiraStanley-Wall, NicolaIvanova, Magdalena I.Stansfield, IanIwata-Reuyl, DirkStelzl, UlrichJewell, Jenna L.Stewart, JasonJohnson, Arlen W.Summer, RossKadakowa, Jun-ichiSutherland, TaraKajava, AndreyTammi, MarkkuKamada, YoshiakiTang, LeiKamat, MedhaThedieck, KathrinKawahara, MasahiroThompson, Karl ManleyKellogg, Douglas R.Toby, BrianKirpekar, FinnTorres, Adrian GabrielKirpich, IrinaTõugu, VelloKlassen, RoalndTroppmair, JakobKleinboelting, SilkeTsolis, Renee M.Kmiec, Eric B.Tuorto, FrancescaKnowles, Tuomas P.J.Urbanc, BrigitaKostyn, KamilUshimaru, TakashiKoźlecki, TomaszValensin, DanielaKraft, ClaudineVan Der Jeught, KevinKrebs, Mark R.H.Van Tilbeurgh, HermanKtistakis, NicholasVelivelli, SivaKuchelmeister, KlausWeisman, RonitKunji, Edmund R.S.Westhof, EricKurta, RuslanWhitford, Paul C.Kwan, AnnWilliams, RogerLanderos-Martínez, LindaWittung-Stafshede, PernillaLandreh, MichaelWondrak, Georg T.Laurenti, EnzoXie, WeiLee, Jun HeeYan, ShengminLegleiter, JustinYeste, MarcLeitinger, BirgitYuchi, ZhiguangLescrinier, EvelineZahan, MariusLimbach, PatrickZallot, Remi G.Lin, RanZhang, GongyiLipke, Peter N.Zhou, ZhanxiangLiu, Chia-ChiZoncu, Roberto


